# 7-Day Nonbismuth-Containing Concomitant Therapy Achieves a High Eradication Rate for *Helicobacter pylori* in Taiwan

**DOI:** 10.1155/2012/463985

**Published:** 2012-07-22

**Authors:** Sung-Shuo Kao, Wen-Chi Chen, Ping-I Hsu, Kwok-Hung Lai, Hsien-Chung Yu, Hui-Hwa Cheng, Nan-Jing Peng, Chiun-Ku Lin, Hoi-Hung Chan, Wei-Lun Tsai, Huay-Min Wang, Tzung-Jiun Tsai, Kung-Hung Lin, Feng-Woei Tsay

**Affiliations:** ^1^Division of Gastroenterology, Department of Internal Medicine, Kaohsiung Veterans General Hospital, Kaohsiung 813, Taiwan; ^2^Department of Pathology, Kaohsiung Veterans General Hospital, Kaohsiung 813, Taiwan; ^3^Department of Nuclear Medicine, Kaohsiung Veterans General Hospital, Kaohsiung 813, Taiwan

## Abstract

*Background.* Ten-day concomitant therapy achieves a high eradication rate in Taiwan. Whether shortening the duration of concomitant therapy can still keep a high eradication rate remains unclear. *Aim.* To assess the eradication rate of 7-day pantoprazole-containing concomitant therapy in Taiwan and to investigate factors influencing the eradication outcome. *Methods.* From March 2008 to March 2012, 319 *H. pylori*-infected patients receiving a 7-day pantoprazole-containing concomitant regimen (pantoprazole 40 mg, amoxicillin 1 g, clarithromycin 500 mg, and metronidazole 500 mg twice daily for 7 days) were included. Patients were asked to return at the second week to assess drug compliance and adverse effects. Repeated endoscopy or urea breath test was performed at 8 weeks after the end of eradication therapy. *Results.* The eradication rates according to intention-to-treat and per-protocol analyses were 93.7% (299/319) and 96.4% (297/308), respectively. Adverse events occurred in 13.2% (42/319) of the patients. The compliance rate was 98.4% (314/319). Multivariate analysis disclosed that poor compliance was the only independent factor influencing the efficacy of anti-*H. pylori* therapy with an odds ratio of 0.073 (95% confidence interval, 0.011–0.483). *Conclusion.* 7-day concomitant therapy achieved a very high eradication rate for *H. pylori* infection in Taiwan. Drug compliance was the only clinical factor influencing treatment efficacy.

## 1. Introduction

*Helicobacter pylori (H. pylori)* infection is a global human pathogen and plays a cardinal role in the development of peptic ulcer disease, gastric adenocarcinoma, and mucosa-associated tissue lymphoma [1]. The Maastricht III Consensus Report has recommended that proton-pump-inhibitor- (PPI-) clarithromycin-amoxicillin or metronidazole treatment for 7 to14 days is the first choice treatment for *H. pylori *infection [2]. Initial data suggested that high eradication rates could be achieved [3, 4]. However, this gold standard has recently become declining in cure rates to unacceptable levels (≦80%), largely as a result of emerging resistance of the organism to clarithromycin [5–9]. In some European countries, the success rates were astonishingly low with values 25~60% [7, 10, 11]. The cure rates for first-line 7-day triple therapy in southern Taiwan declined from 84% to 80% in recent 5 years [4, 12]. Therefore, searching for more effective first-line therapies is urgently required [4, 13].

One recent therapeutic innovation is 10-day sequential regimen with a 5-day dual therapy (a PPI plus amoxicillin), followed by a 5-day triple therapy (a PPI plus clarithromycin and tinidazole (or metronidazole)) [14]. Several studies have demonstrated its satisfactory higher eradication rates than standard triple therapies [8, 9, 15]. Gatta et al. reported a rigorous systemic review that identified 13 trials evaluating 3,271 patients [16]. Most of the studies were conducted in Italy, where the patterns of clarithromycin and metronidazole resistance tend to be similar to those in United States and Europe. The data show that sequential therapy achieves 90.7% eradication rates, with a 12% better absolute eradication rate than the standard triple therapy [16]. Our recent study also demonstrated that sequential therapy achieved a higher eradication rate than standard triple therapy in Taiwan (93% versus 80%, *P* = 0.005) [12]. However, a trial from Korea revealed that both sequential therapy and triple therapy achieved similar efficacy with unsatisfactory eradication rates (85.7% versus 76.6%, by PP analysis, *P* = 0.150) [17]. Generally speaking, sequential therapy is a good but typically not an excellent regimen (i.e., typically achieving a grade B and not grade A result) [18]. Theoretically, sequential therapy can be improved [19].

Concomitant therapy uses the same components as sequential therapy, but they are administered concomitantly [20]. It provides another novel regimen proven successful in the presence of clarithromycin resistance [21]. It is a 4-drug regimen containing a PPI (standard dose, b.i.d.), clarithromycin (500 mg, b.i.d.), amoxicillin (1 g, b.i.d.), and metronidazole (500 mg, b.i.d.) which are all given for the entire duration of therapy [22, 23]. This approach achieved more than 90% of eradication rates. A head-to-head noninferiority trial of 10-day esomeprazole-containing concomitant and 10-day esomeprazole-containing sequential therapy from our study group showed they were equivalent (93.0% versus 93.1% by per-protocol analysis) [24]. Nonetheless, a large-scaled, randomized controlled trial from Latin America revealed that the per-protocol eradication rates of 14-day lansoprazole-containing standard triple, 5-day concomitant and 10-day sequential therapies were 87%, 79%, and 81%, respectively [25]. The 5-day lansoprazole-containing concomitant therapy and 10-day sequential therapy had comparable eradicate rates. However, the eradication rate of 5-day concomitant therapy was lower than that of 14-day standard triple therapy. The insufficient treatment duration of concomitant therapy in the study was possibly an important factor accounting to the unacceptable eradication rate (< 80%) of the new therapy.

Currently, the optimal duration of concomitant therapy is unknown, and whether shortening the duration of concomitant therapy from 10 days to 7 days can still keep a high eradication rate for *H. pylori *infection remains unclear. In this study, we retrospectively assess the eradication rate of 7-day nonbismuth containing concomitant therapy in Taiwan and investigated the host factors influencing the eradication outcome.

## 2. Materials and Methods

### 2.1. Patients

From March 2008 to March 2012, 319 *H. pylori*-infected outpatients who received a 7-day pantoprazole-containing concomitant therapy in our center were included for the retrospective analysis. The exclusion criteria included (a) previous eradication therapy, (b) consumption of antibiotics, bismuth, or proton pump inhibitors within previous 4 weeks, (c) allergy to antibiotics or PPIs, (d) patients with previous gastric surgery, (e) the coexistence of serious concomitant illness (such as, decompensated liver cirrhosis, uremia), and (f) pregnant women. The *H. pylori* infection was defined by at least one positive result of following: culture, rapid urease test, histology, or urea breath test.

### 2.2. Study Design

In the study period, 317 *H. pylori*-infected patients received a 7-day pantoprazole-containing concomitant therapy (pantoprazole 40 mg, clarithromycin 500 mg, amoxicillin 1 g and metronidazole 500 mg twice daily). According to the standard protocol for *H. pylori* eradication therapy in our institute, all drugs were taken one hour before breakfast and dinner. Patients were asked to return at the second week to assess drug compliance and adverse effects. Repeated endoscopy with rapid urease test and histological examination or urea breath test was performed at 8 weeks after the end of anti-*H. pylori *therapy. Successful eradication was defined as (a) negative results of both rapid urease test and histology in follow-up endoscopy, or (b) a negative result of urea breath test. 

### 2.3. Questionnaire

A complete medical history and demographic data were obtained from each patient, including age, sex, medical history, history of smoking, alcohol, coffee, and tea consumption. Adverse events were prospectively evaluated. The adverse events were retrospectively assessed according to a 4-point scale system: none; mild (discomfort annoying but not interfering with daily life); moderate (discomfort sufficient to interfere with daily life); and severe (discomfort resulting in discontinuation of eradication therapy) [26]. Compliance of patients was checked by counting unused medication at the completion of treatment. Poor compliance was defined as taking less than 80% of the total medication.

### 2.4. Rapid Urease Test, Histology, and Urea Breath Test

The rapid urease test, histology, and urea breath test were performed according to our previous studies [13]. A biopsy specimen was taken from the lesser curvature site of the antrum for rapid urease test. Two biopsy specimens were each taken from the lesser curvature sites of the antrum and the corpus for histological examination [26]. The cut-off value of urea breath test was set at 4.8% of *δ* ^13^CO_2_ [27]. 

### 2.5. Statistical Analysis

The primary outcome variables were the rates of eradication, adverse events, and compliance. The overall eradication rates and their 95% confidence intervals were obtained by ITT and per protocol (PP). ITT analysis included all patients who had taken at least one dose of study medication. Patients whose infection status was unknown following treatment were considered treatment failures for the purposes of ITT analysis. The PP analysis excluded the patients with unknown *H. pylori *status following therapy and those with poor compliance.

To determine the independent factors affecting the treatment response, 11 clinical and endoscopic parameters were analyzed by univariate analysis. These variables included the following: age (<60 or ≥60 years); gender; history of current smoking (<1 pack/week or ≥1 pack/week), history of current alcohol consumption (<80 g/day or ≥80 g/day), ingestion of coffee (<1 cup/day or ≥1 cup/day), ingestion of tea (<1 cup/day or ≥1 cup/day), coexistence of a systemic disease (yes or no); previous history of peptic ulcer disease, endoscopic appearance (ulcer or gastritis), types of PPI, and drug compliance (good or poor). Chi-square test with or without Yates correction for continuity and Fisher's exact test were used when appropriate to compare the treatment outcome and host factors using the SPSS program (version 10.1, Chicago, IL, USA). A *P* value less than 0.05 was considered statistically significant. Those variables found to be significant by univariate analysis were subsequently assessed by a stepwise logistic regression method to identify independent factors for eradication outcome. 

## 3. Results

### 3.1. Patients

A total of 319 patients received concomitant therapy from March 2008 to March 2012. The subjects were all included in the ITT analysis for *H. pylori* eradication. Data regarding the clinical characteristics of patients at entry are summarized in Table 1. Among the subjects, five with poor compliance and six with incomplete followup were excluded from PP analysis for *H. pylori *eradication. All patients were included in intention-to-treat analysis.

### 3.2. Eradication of *H. pylori*

Table 2 lists the therapeutic outcomes of the 7-day concomitant therapy. According to the ITT analysis,* H. pylori *infection was eradicated in 93.7% (299/319) of the patients receiving concomitant therapy. By PP analysis, the treatment rate was 96.4% (297/308). Five of 319 patients (1.6%) failed to complete the treatment because of insufficient compliance. *H. pylori* was successfully eradicated in three of 5 cases (60%). 

### 3.3. Adverse Effect and Compliances

All of the 319 patients were included in the adverse event analysis. In total, 13.2% (42/319) of the patients reported at least one adverse event during eradication therapy. The profiles and frequencies of adverse events were listed in Table 3. The most frequent symptoms were nausea (26 patients; 8.2%) and headache (11 patients; 3.4%). Less-frequent symptoms were abdominal pain (3 patients; 0.9%), abdominal constipation (2 patients; 0.6%), and diarrhea (2 patients; 0.6%). There were 5 patients who discontinued treatment as a result of adverse events during eradication therapy (nausea: 2 patient; headache: 2 patients; diarrhea: 1 patient). Overall, the compliance rate was 98.4%.

### 3.4. Factors Influencing Efficacy of Anti-*H. pylori* Therapy

Table 4 lists the clinical and endoscopic factors influencing the efficacy of eradication therapy. Only patients with known follow-up *H. pylori *status (*n* = 312) were included for the analysis. The eradication rates were significantly related to drug compliance (*P* = 0.015) and smoking (*P* = 0.046) in univariate analysis. The other factors (age, sex, alcohol drinking, coffee consumption, tea consumption, previous history of ulcer, presence of ulcer, and presence of adverse event) did not markedly influence the eradication efficacy. Multivariate analysis disclosed that poor compliance was the only independent factor influencing the efficacy of anti-*H. pylori* therapy (Table 5). The odds ratios were 0.073 (95% confidence interval (CI), 0.011–0.483).

## 4. Discussion

The fall in *H. pylori* eradication rates with standard triple therapies resulted in a search for novel therapies for* H. pylori* infection [6]. In this study, we examined the efficacy of 7-day pantoprazole-containing concomitant therapy in Taiwan. We found that the 7-day concomitant therapy produced 96.4% treatment success by PP analysis. The eradication rate by ITT analysis was 93.7%. In our two previous studies [4, 12], the eradication rates of 7-day pantoprazole-containing standard triple therapy by ITT analysis were 84% and 80%, respectively. The data suggested that 7-day concomitant therapy achieved a high eradication rate in Taiwanese and had a great potential to replace 7-day triple therapy as a first-line anti-*H. pylori* therapy in Taiwan.

In the initial studies from Germany and Japan for concomitant therapy, a PPI and three antibiotics (amoxicillin, clarithromycin, and metronidazole) for 5–7 days achieved high eradication rates [22, 23]. A meta-analysis published in 2009 presented the pooled eradication rate of concomitant therapy studies between 1998 to 2002 as 89.7% on ITT and 92.9% on PP analysis [20]. In recent years, concomitant therapies with duration of 5–10 days are reported 90–96% success rates on PP analysis in Asian countries, including Thailand, Taiwan, and Korea [24, 28, 29]. The high eradication rate (94.5%) was also reported in Europe, such as Greece [30]. However in Latin America, Greenberg et al. pointed that the success rate of 5-day concomitant therapy was dropped to 78.7% [25]. A recent review article of 15 studies (1723 patients) revealed that there was a tendency towards better results by longer treatments (7–10 days versus 3–5 days) [21]. Our previous study showed that 10-day esomeprazole- containing concomitant therapy achieved a 93% eradication rate [24]. In the current study, we shortened the duration of concomitant therapy from 10 days to 7 days, and still achieved high eradication rates. The results indicated that the duration of concomitant therapy could be shortened to 7 days in Taiwan.

In this investigation, 13% of the patients treated with concomitant therapy reported at least one adverse event during eradication therapy. Basically, concomitant therapy was well tolerated and had good compliance (98.4%). The most frequent symptoms were nausea (8%) and headache (3%). Only five patients discontinued treatment as a result of adverse events during eradication therapy. Multivariate analysis revealed that drug compliance was the only independent clinical factor influencing treatment efficacy. The eradication rates in patients with good and poor compliance were 96.4% and 60%, respectively. Notably, the occurrence of severe adverse events was an important cause of poor drug compliance.

Smoking has been shown to reduce the effectiveness of first line triple therapy [31]. However, in recent Taiwanese study, no significant effect of smoking was found in concomitant and sequential therapy [24]. In this study, smoking is one of clinical factors influencing treatment outcome by univariate analysis. The reason for this is possibly due to low prevalence rate of smoking. However, in multivariate analysis, it is no longer an independent factor.

This study has several limitations. Firstly, it was a retrospective study, although all patients were prospectively followed up by a standard protocol and the adverse events and compliance were assessed by trained assistants with study nurses with a standardized questionnaire. Secondly, the impacts of antibiotic resistance on the eradication rate could not be assessed by the study because a routine culture was not conducted in the first-line therapy. However, this study is the first work to investigate 7-day pantoprazole-containing concomitant therapy and the number of cases in this study was large (*n* = 319). Thirdly, in tradition, subjects need to have both positive tests of urease test and histology or positive result of culture to be counted as infected for entry into a clinical trial. In this study, we enrolled the patients with one positive test for *H. pylori* infection. The eradication rates may be overestimated since patients with false-positive *H. pylori* infection achieve successful eradication in the trial. But generally false-positive tests for urease test are uncommon [32]. Therefore, this study represents the real world scenario for *H. pylori* detection and eradication.

In conclusion, 7-day concomitant therapy achieved a very high eradication rate for *H. pylori* infection in Taiwan. It was well tolerated. Drug compliance was the only clinical factor influencing treatment efficacy. 

## Figures and Tables

**Table 1:** Demographic data and endoscopic appearance of 7-day concomitant therapy.

Characteristics	7-day concomitant therapy group (*n* = 319)
Age (yr) (mean ± SD)	53 ± 12
Gender (male/female)	189/130
Smoking	71 (22.3%)
Alcohol consumption	24 (7.5%)
Ingestion of coffee	81 (25.4%)
Ingestion of tea	124 (38.9%)
NSAID use	16 (5%)
Underlying disease	80 (25.1%)
Endoscopic findings	
Gastritis	98 (30.7%)
Gastric ulcer	100 (31.3%)
Duodenal ulcer	56 (17.6%)
Gastric ulcer + duodenal ulcer	65 (20.4%)

**Table 2:** The major outcomes of 7-day concomitant therapy.

	Outcome of 7-day concomitant therapy (*n* = 319)
Eradication rate	
Intention to treat	93.7% (299/319)
Per protocol	96.4% (297/308)
Adverse events	13.2% (42/319)
Compliance	98.4% (314/319)

**Table 3:** Adverse events of 7-day concomitant therapy.

Adverse events	7-day concomitant therapy group (*n* = 319)
Abdominal pain	3 (1/1/1^∗^)
Constipation	2 (1/0/1)
Diarrhea	2 (1/0/1)
Dizziness	0 (0/0/0)
Headache	11 (7/2/2)
Nausea/vomiting	26 (14/9/3)
Taste perversion	5 (3/1/1)
Palpitation	3 (2/1/0)
Insomnia	3 (1/1/1)
Other	21 (13/4/4)

^∗^ Number of patients who suffered from mild, moderate, and severe adverse events.

**Table 4:** Univariate analysis of the clinical factors influencing the efficacy of *H. pylori* eradication therapy.

Principle parameter	No. of patients	Eradication rate	*P* value
Age			0.762
<60 years	220	95.5%	
≧60 years	92	96.7%	
Sex			0.010
Female	129	92.2%	
Male	183	98.4%	
Smoking			0.046
(−)	242	94.6%	
(+)	70	100%	
Alcohol consumption			0.610
(−)	290	95.5%	
(+)	22	100%	
Ingestion of coffee			0.294
(−)	231	96.5%	
(+)	81	93.8%	
Ingestion of tea			0.085
(−)	189	94.2%	
(+)	123	98.4%	
NSAID use			0.503
(−)	296	95.9%	
(+)	16	93.8%	
Previous history of peptic ulcer			
(−)			
(+)			
Presence of ulcer			0.314
(−)	65	94.9%	
(+)	234	95.1%	
Compliance			0.015
(−)	5	60%	
(+)	307	96.4%	
Side effect			0.082
(−)	270	96.7%	
(+)	42	90.5%	

**Table 5:** Multivariate analysis for clinical factors related to eradication efficacy of *H. pylori. *

Clinical factor	Coefficient	Standard error	Odds ratio (95% CI)	*P* value
Poor compliance	−2.617	0.964	0.073 (0.011–0.483)	0.007
